# Obesity and Cardiovascular Disease: Mechanistic Insights and Nutritional Management Strategies

**DOI:** 10.3390/nu17142281

**Published:** 2025-07-10

**Authors:** Ehud Grossman

**Affiliations:** Adelson School of Medicine, Ariel University, Ariel 4077625, Israel; ehud.grossman@ariel.ac.il; Tel.: +972-39066153

Obesity and overweight represent a growing global epidemic. As of 2021, an estimated 1.0 billion men and 1.1 billion women over the age of 25 were affected by overweight and obesity. Projections suggest that by 2050, more than half of the world’s adult population—approximately 3.8 billion individuals—will be classified as overweight or obese [1]. A recent study further reported that between 1990 and 2021, the combined prevalence of overweight and obesity in children and adolescents doubled, while the prevalence of obesity alone tripled [2].

Globally, the rise in obesity is driven by a convergence of factors, including sedentary behavior, energy-dense diets, and broader socioeconomic influences. Obesity is associated with reduced life expectancy and increased risk for numerous comorbidities, including type 2 diabetes, hypertension, dyslipidemia, non-alcoholic fatty liver disease (NAFLD), cardiovascular diseases (e.g., myocardial infarction, heart failure, atrial fibrillation, stroke), obstructive sleep apnea, osteoarthritis, and certain cancers [3]. While BMI is widely used to define obesity, it is now well recognized that central (abdominal) adiposity is a more accurate predictor of cardiometabolic risk. Anthropometric measures such as waist circumference, waist-to-hip ratio, and waist-to-height ratio offer better estimates of visceral fat burden [3,4].

Adipose tissue functions not merely as an energy storage depot but as a metabolically active endocrine organ. It secretes various adipokines—such as leptin, adiponectin, and resistin—that influence inflammation, insulin sensitivity, and vascular homeostasis [3]. Dysregulation of these signals can lead to endothelial dysfunction and a chronic pro-inflammatory state, which are key contributors to atherosclerosis.

In individuals with obesity, adipose tissue is marked by hypertrophic adipocytes, ectopic fat deposition, dysregulated adipocytokine profiles, and the induction of chronic low-grade inflammation and fibrosis. These processes contribute to insulin resistance and the eventual development of type 2 diabetes [3].

Obesity is a well-established risk factor for hypertension [5]. Several mechanisms may mediate this link, including increased sympathetic nervous system activity, activation of the renin–angiotensin–aldosterone system, insulin resistance with sodium retention, and elevated leptin levels [3,6]. Weight loss has been shown to improve blood pressure control through the reversal of these mechanisms [7,8]. However, its benefits depend on sustained weight reduction; even modest weight regain can negate the improvements [9]. Shlomai et al. showed that minor, unintentional weight gain can lead to elevated blood pressure [10].

Obesity is also associated with ectopic fat accumulation in various organs. Hepatic steatosis is a well-documented consequence, and NAFLD is closely linked to BMI and waist circumference [11]. Emerging data indicate that fat accumulation in the kidneys may also occur. Importantly, renal fat accumulation may induce inflammation and fibrosis, serving as a potential early marker for chronic kidney disease (CKD) development in patients with obesity. Renal steatosis has also been associated with diabetes, CKD progression, and hypertension, and its early identification may have important clinical implications for risk stratification and prevention [12].

Management of obesity-associated cardiovascular disease involves both aggressive control of cardiometabolic risk factors and implementation of weight reduction strategies. Risk factor management focuses on blood pressure, glucose, and lipid control, along with smoking cessation [3].

Lifestyle modification, including a calorie-restricted carbohydrate-reduced diet and regular physical activity, is recommended for all individuals with obesity. These interventions can be effective in achieving weight loss. For patients unable to maintain such changes or who do not respond adequately, pharmacological therapy may be appropriate [3].

Recent advances have shown that GLP-1 receptor agonists (e.g., semaglutide, orforglipron) and dual GLP-1/GIP receptor agonists (e.g., tirzepatide) not only lower glucose levels but also result in significant weight loss, improved metabolic parameters, and reduced cardiovascular events [13,14,15]. SGLT2 inhibitors may also support modest weight loss and confer cardiovascular protection [3,16].

In cases of severe obesity, or when pharmacologic treatments are poorly tolerated or ineffective, bariatric surgery should be considered. These procedures are associated with substantial and sustained weight loss, metabolic improvement, and reduction in cardiovascular morbidity [3,17,18].

This Issue offers a comprehensive overview of the multifaceted relationship between obesity and cardiovascular disease. It addresses diagnostic approaches, nutritional and environmental etiologies, the renal consequences of obesity, and the impact of behavioral and weight changes on cardiometabolic risk.
